# In vivo estrogenicity of *p*-phenoxyphenol and *p*-pentyloxyphenol

**DOI:** 10.1038/s41598-020-73271-1

**Published:** 2020-10-14

**Authors:** Yue Wang, Han Xiao, Lei Yang, Xiaojing Jia, Xuan Guo, Zhaobin Zhang

**Affiliations:** College of Urban and Environmental Sciences, MOE Laboratory for Earth Surface Processes, Peking University, Beijing, 100871 China

**Keywords:** Environmental sciences, Environmental impact

## Abstract

*p*-Alkoxyphenols (AOPs) are a class of ethers that are widely used in industrial and agricultural productions and daily necessities. *p*-Phenoxyphenol (PhOP) and *p*-pentyloxyphenol (PeOP) belong to this class and have been reported to be estrogenic in vitro. However, their in vivo estrogenic activities have rarely been of concern. In this study, we performed an immature mouse uterotrophic assay and studied the estrogenic effects of these two compounds in mice. The results revealed that the uterine weights of the animals treated with PhOP significantly increased at doses of 30 and 300 mg kg^-1^ bw day^-1^ for 3 days (*P* < 0.05), while no significant uterotrophic effects were observed in the mice treated with PeOP. Using next-generation transcriptome sequencing (RNA-seq), we also analyzed the gene expression in the uterine tissue of mice treated with PhOP and PeOP. The observed gene regulation patterns of the PhOP- and PeOP-treated specimens were similar to those of the 17*β*-estradiol (E_2_)-treated specimens. In particular, some estrogen-responsive genes, such as the *Sprr2* gene family, *Apoa1*, *Prap1*, and *Ahsg*, displayed a regulation trend similar to that of E_2_. In addition, molecule docking analysis revealed that both PhOP and PeOP could be well docked into the active site of hERα, with potential of mean force (PMF) values of − 58.68 and − 52.67 kcal mol^-1^ for PhOP and PeOP, respectively. The results of this study indicate that PhOP exhibits relatively strong in vivo estrogenic activity, which could be of future concern.

## Introduction

Environmental estrogens (EEs) are a class of xenobiotics that act as natural estrogens and disrupt the synthesis, release, secretion, receptor-binding, and metabolism of endogenous estrogens in the body^1^. Over the past decades, studies have shown that EEs can affect the reproductive system, central nervous system, pancreas, and immune system and cause a wide range of diseases such as precocious puberty, breast hyperplasia, spontaneous abortion, and reproductive system cancers^2–5^. However, to date, the identified EEs are still the tip of the iceberg^6^, and only a small fraction of these synthetic chemicals has been tested for their potential estrogenic properties. In recent years, high-throughput screening for EEs has been performed via in vitro assays and some chemicals have been screened as potential EEs. Verification and evaluation of these potential EEs by in vivo toxicity tests have become priorities of EE studies.

**Figure 1:**
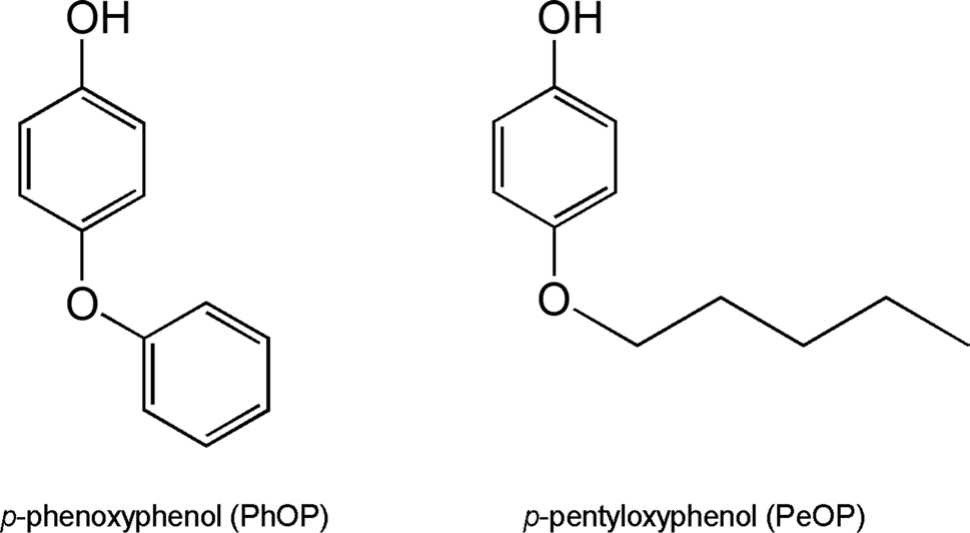
Chemical structures of *p*-phenoxyphenol (PhOP) and *p*-pentyloxyphenol (PeOP).

*p*-Alkoxyphenols (AOPs) are a class of ethers comprising phenol structures that are similar to some typical EEs such as alkylphenols. Several in vitro studies have shown that among the AOPs, *p*-phenoxyphenol (PhOP, CAS: 831-82-3, C_12_H_10_O_2_) and *p*-pentyloxyphenol (PeOP, CAS: 18979-53-8, C_11_H_16_O_2_) possess stronger estrogenic activities^7–9^. However, there are no available data on the in vivo estrogenic activities of these two compounds, even though they have been listed in the TSCA Chemical Substance Inventory^10^. PhOP is used as a material for the synthesis of pesticides, such as pyriproxyfen and fenoxycarb^11^; it is also used in the manufacture of heat-sensitive recording material^12^, phenolic resin^13^, polymers^14^, polyamides^15^, pharmaceutical compounds^16–18^, etc. The annual production of PhOP in United Kingdom was reported to be about 30 tons in 2007^19^. Studies have shown that PhOP can be released as a monomer or metabolite from PhOP-containing products^20–22^. Masao et al. (2005) investigated the metabolism of the insect growth regulator pyriproxyfen in tomatoes plants and found that PhOP was detectable in tomato fruit as a metabolite. Moreover, the in vitro metabolism study of pyriproxyfen using hepatic microsomes of mice and rats demonstrated that PhOP is also a pyriproxyfen metabolite in animals^22^. Besides, it was found that PhOP was included in atmosphere organic aerosols emerging from biomass burning^23^. As for PeOP, there are few studies of its pollution. PeOP is usually used as a material in the synthesis of products including bactericides^24^, cosmetics^25^, fragrances^26^, liquid crystal intermediates^27^, phenolic resin^28^, and styrene polymers^29^. The structures of PhOP and PeOP are shown in Fig. 1.

Notably PhOP and PeOP, are widely used in industrial and agricultural productions and daily necessities, and PhOP monomers may be released from PhOP-containing products as metabolites and degradants. Thus, because humans could become inevitably exposed to these chemicals, it is necessary to study the in vivo estrogenicity of these two compounds.

## Results and discussions

### Uterotrophic effects of PhOP and PeOP in immature CD-1 mice

**Figure 2:**
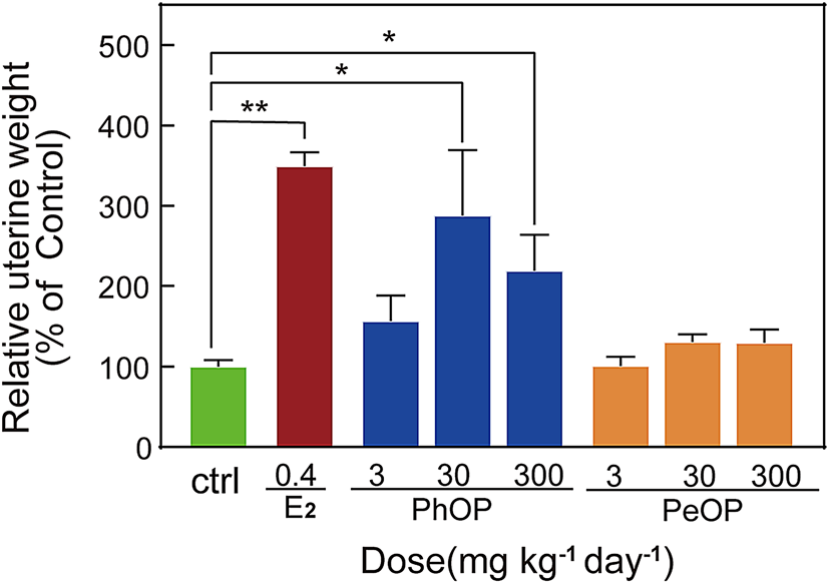
Uterotrophic effects of *p*-phenoxyphenol (PhOP) and *p*-pentyloxyphenol (PeOP) in immature CD-1 mice. All the treated mice (n = 8) were administered three days of oral gavage from PND 21 and the relative uterine weight was calculated. Data were expressed as percentages of control treated and the error bars indicated the standard errors of mean (SEMs) from the average values; *P < 0.05; **P < 0.01.

The uterotrophic assay is a useful in vivo approach to determine the estrogenic activities of chemicals^30^ and has been incorporated into the USEPA screening and testing program for EEs^31^. To better understand the PhOP and PeOP estrogenic activities, we performed an uterotrophic assay using immature CD-1 mice to identify their in vivo estrogenic potentials. There was no mortality during the treatment period. Our previous in vitro study showed that the estrogenic activities of PhOP and PeOP were about 2–3 orders of magnitude lower than that of E_2_^8^. So we selected doses of 1–3 orders of magnitude higher than E_2_ for uterotrophic assay. Figure 2 reveals an increase in the relative uterine weights of the CD-1 mice in all the test groups as compared to that of the mice in the vehicle control group. The relative uterine weights of the mice treated with E_2_ (0.4 mg kg^-1^ bw day^-1^) increased to 349% that of the control (*P* < 0.01), indicating that the animals and experiment were reliable. In the mice treated with 30 and 300 mg kg^-1^ bw day^-1^ doses of PhOP, the relative uterine weights were significantly (*P* < 0.05) increased to 287 and 218% that of the control, respectively. In the groups treated with 3 mg kg^-1^ bw day^-1^ PhOP, the relative uterine weights also increased to 157% that of the control; however, no statistical significance was observed when compared to the control results (*P* > 0.05). In the PeOP-treated mice, the average uterine weights increased in a dose-dependent pattern and again, no statistical significance (*P* > 0.05) was observed when compared to the control results. The results of the uterotrophic assay indicated that PhOP exhibited marked estrogenic activity in animals at low doses, while the in vivo estrogenic activity of PeOP was weak. These observations were consistent with previously published in vitro data^7^, whereby the PhOP estrogenic activity in a recombinant yeast assay was reported to be ~ 66-fold higher than that of PeOP^7^. Several alkylphenol chemicals are known as classic environmental estrogens and have been reported to have uterotrophic effects in immature rodents. *p*-Nonylphenol at doses of 100 and 200 mg kg^-1^ bw day^-1^ has been reported to significantly increase the uterine weights of the treated rats^32^; *p*-cyclohexylphenol and *p*-(phenylmethyl)phenol at dose of 200 mg kg^-1^ bw day^-1^ have significantly increased the uterine weights of the treated rats, respectively; and *p*-amylphenol at dose of 800 mg kg^-1^ bw day^-1^ have been reported to significantly increase the uterine weights of the treated rats^33^. The in vivo estrogenicity of PhOP observed in this study is similar to that of the alkylphenols.

### Transcriptome analysis of mice uteri treated with PhOP and PeOP

**Figure 3:**
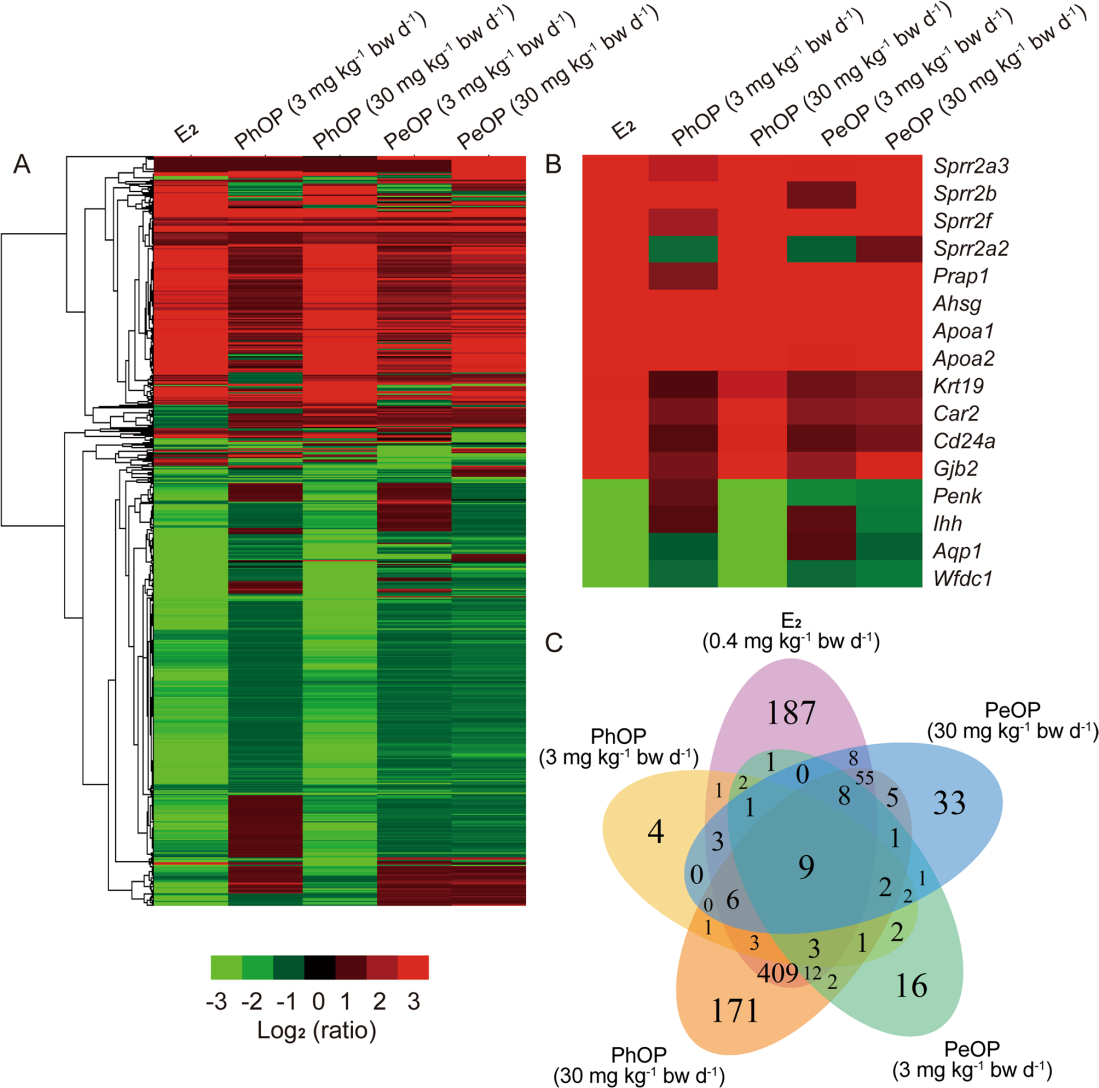
Analyses of the differentially expressed genes (DEGs): **(A)** cluster analysis of the DEGs in the 17*β*-estradiol (E_2_)-, *p*-phenoxyphenol (PhOP) and *p*-pentyloxyphenol (PeOP)-treated groups; **(B)** heatmap of the estrogen-responding marker genes; and **(C)** Venn diagram of the DEGs in the E_2_-, PhOP-, and PeOP-treated groups.

RNA-seq is a useful tool to study the mechanisms underlying the toxicity of chemicals in vivo^34^. In this study, we used RNA-seq to analyze the transcriptome of mice uteri treated with PhOP or PeOP at dosages of 3 and 30 mg kg^-1^ bw day^-1^. DEGs as well as DEG-enriched GO terms and KEGG pathways^35–37^ were also studied. Figure 3A illustrates the gene regulation profiles by E_2_, PhOP, and PeOP. The most evident feature in the gene profiles was that the DEG regulation patterns in the uteri of the PhOP- and PeOP-treated mice were similar to that of the E_2_ group. Out of all the DEGs, some genes that have been previously reported as estrogen-response markers were of great concern. The small proline-rich 2 (*Sprr2*) gene family comprises 11 genes that encode the cross-linked envelope proteins of keratinocytes. The *Sprr2* genes were reported to be positively regulated by estrogen receptor-dependent pathways in the mice uteri^38^. In this study, the *Sprr2* family genes, including *Sprr2a2*, *Sprr2a3*, *Sprr2b*, and *Sprr2f*, were significantly up-regulated by PhOP and PeOP at the dose 30 mg kg^-1^ bw day^-1^. Of them, *Sprr2b* was the most sensitive gene with fold changes of 183 in the E_2_ group, 105 in the 30 mg kg^-1^ bw day^-1^ PhOP group, and 16 in the 30 mg kg^-1^ bw day^-1^ PeOP group. Apolipoprotein A I (*Apoa1*) encodes the major protein of plasma high density lipoprotein^39^. Study has revealed that estrogens induce Apoa1 gene expression^40^. In this study, *Apoa1* was significantly up-regulated by PhOP and PeOP with fold changes of 142 in the E_2_ group, 80 in the 3 mg kg^-1^ bw day^-1^ PhOP group, 85 in the 30 mg kg^-1^ bw day^-1^ PhOP group, 8 in the 3 mg kg^-1^ bw day^-1^ PeOP group, and 118 in the 30 mg kg^-1^ bw day^-1^ PeOP group. Proline-rich acidic protein 1 (*Prap1*) was also reported as an estrogen up-regulated gene, which is considered to play a crucial role in the maintenance of gestation^41^. In this study, *Prap1* was also up-regulated by PhOP and PeOP with fold changes of 51 in the E_2_ group, 2 in the 3 mg kg^-1^ bw day^-1^ PhOP group, 57 in the 30 mg kg^-1^ bw day^-1^ PhOP group, 3 in the 3 mg kg^-1^ bw day^-1^ PeOP group, and 16 in the 30 mg kg^-1^ bw day^-1^ PeOP group. In addition, alpha-2-HS-glycoprotein (*Ahsg*), a glycoprotein gene that was up-regulated by estrogen in women^42^, was also up-regulated in the mice uteri in this study with fold changes of 172 in the E_2_ group, 103 in the 3 mg kg^-1^ bw day^-1^ PhOP group, 97 in the 30 mg kg^-1^ bw day^-1^ PhOP group, 9 in the 3 mg kg^-1^ bw day^-1^ PeOP group, and 159 in the 30 mg kg^-1^ bw day^-1^ PeOP group. These expression data of the estrogen-response marker genes indicated that both PhOP and PeOP displayed estrogenic activities at the transcriptome level. The regulation patterns of more estrogen-responsive marker genes are presented in Fig. 3B. The Venn diagram of the gene overlap shows the comparison of the DEG numbers in the uteri of E_2_-, PhOP-, and PeOP-treated mice (Fig. 3C): 708 DEGs in E_2_ group, 39 and 687 DEGs in 3 and 30 mg kg^-1^ bw day^-1^ PhOP groups, and 62 and 133 DEGs in the 3 and 30 mg kg^-1^ bw day^-1^ PeOP groups, respectively.

**Figure 4:**
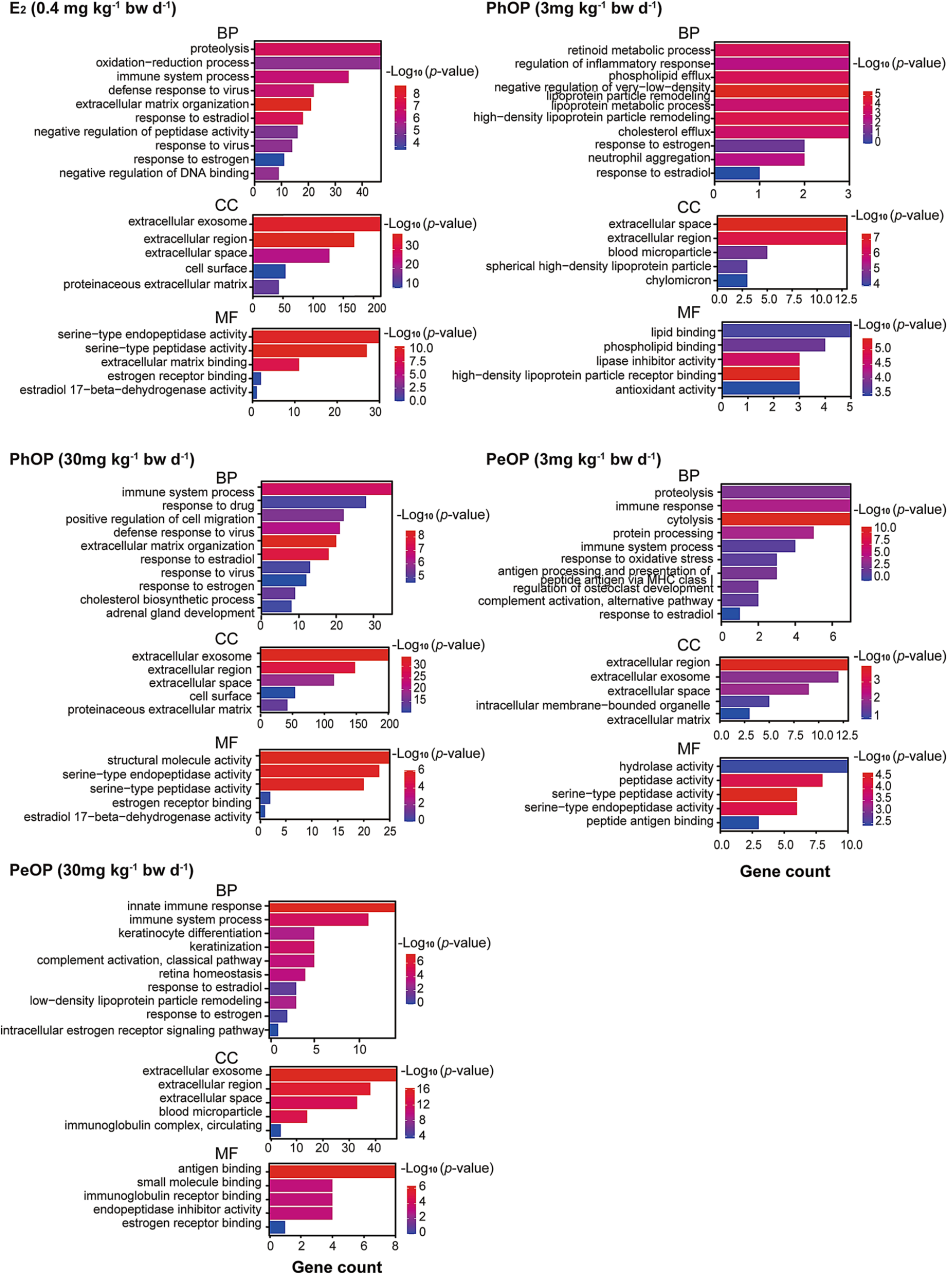
Histograms of the gene ontology (GO) classifications: GO enrichment terms of the differentially expressed genes (DEGs) in 0.4 mg kg^-1^ bw day^-1^ 17*β*-estradiol (E_2_)- treated uteri; 3 and 30 mg kg^-1^ bw day^-1^ dose of *p*-phenoxyphenol (PhOP)- treated uteri; 3 and 30 mg kg^-1^ bw day^-1^ dose of *p*-pentyloxyphenol (PeOP)-treated uteri. BP: biological process, CC: cellular components, MF: molecular functions.

In the GO enrichment analysis, DEGs were annotated to the GO database and enriched in three main categories: biological processes (BP), cellular components (CC), and molecular functions (MF) (Fig. 4). DEGs in the 0.4 mg kg^-1^ bw day^-1^ E_2_ group significantly enriched 304 terms in the BP, 43 terms in the CC, and 78 terms in the MF. DEGs in the 3 mg kg^-1^ bw day^-1^ PhOP group significantly enriched 41 terms in the BP, nine terms in the CC, and 17 terms in the MF. DEGs in the 30 mg kg^-1^ bw day^-1^ PhOP group significantly enriched 263 terms in the BP, 63 terms in the CC, and 87 terms in the MF. DEGs in the 3 mg kg^-1^ bw day^-1^ PeOP group significantly enriched 11 terms in the BP, three terms in the CC, and seven terms in the MF. DEGs in the 30 mg kg^-1^ bw day^-1^ PeOP group significantly enriched 52 terms in the BP, 14 terms in the CC, and 28 terms in the MF. Notably, the “response to estrogen” and the “response to estradiol” in the BP category were enriched by 0.4 mg kg^-1^ bw day^-1^ E_2_ group, 3 and 30 mg kg^-1^ bw day^-1^ PhOP groups, and 30 mg kg^-1^ bw day^-1^ PeOP groups. The “estrogen receptor binding” and the "estradiol 17-beta-dehydrogenase activity" in the MF category were enriched by 0.4 mg kg^-1^ bw day^-1^ E_2_ group and 30 mg kg^-1^ bw day^-1^ PhOP group.

**Figure 5:**
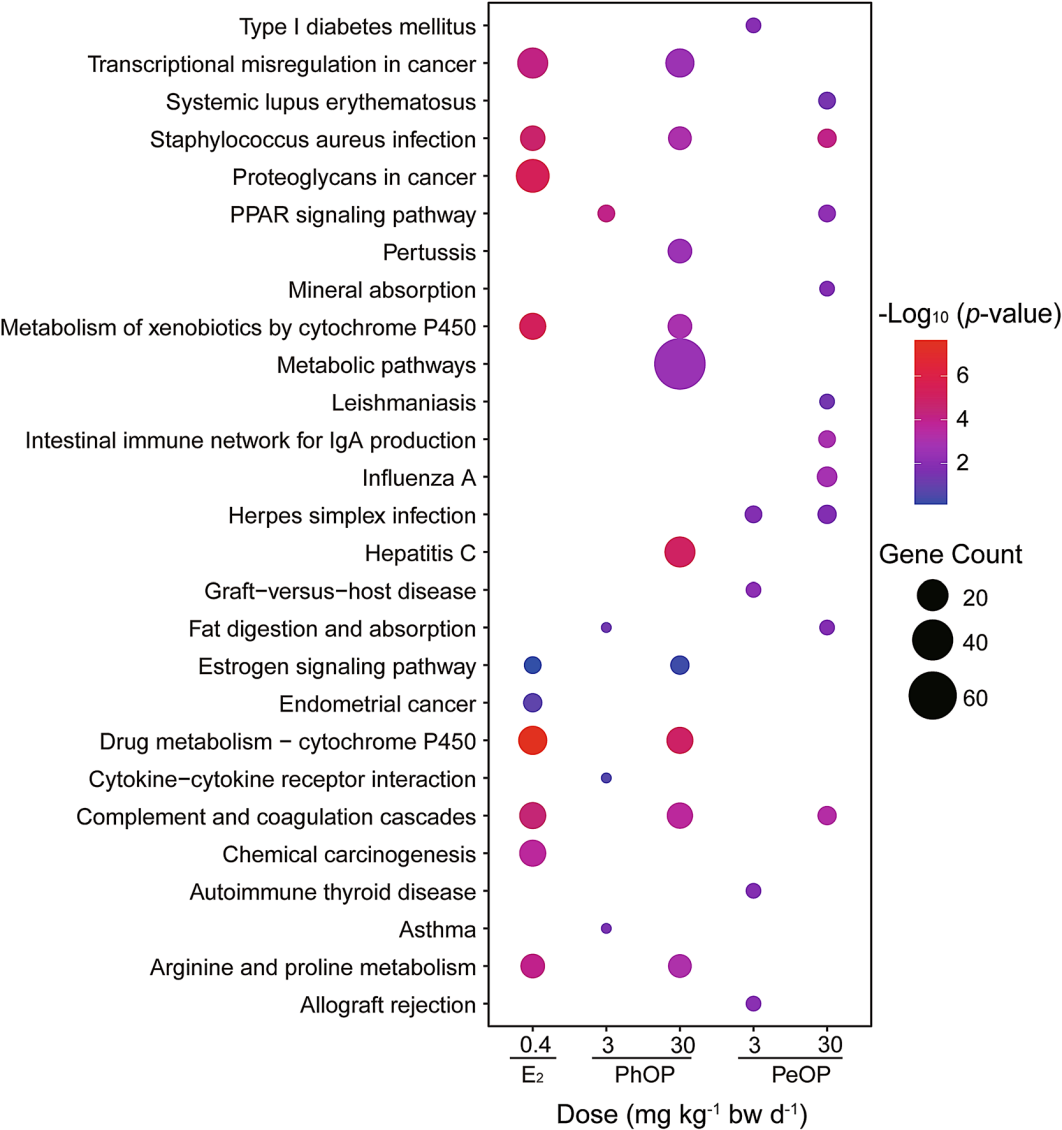
Kyoto Encyclopedia of Genes and Genomes (KEGG) pathway analysis of the differentially expressed genes (DEGs) in mice uteri treated with 17*β*-estradiol (E_2_; 0.4 mg kg^-1^ bw day^-1^), *p*-phenoxyphenol (PhOP; 3 and 30 mg kg^-1^ bw day^-1^), and *p*-pentyloxyphenol (PeOP; 3 and 30 mg kg^-1^ bw day^-1^).

KEGG pathway analysis was also performed to determine the pathways in which the DEGs were significantly enriched (*P* < 0.05). DEGs in the 0.4 mg kg^-1^ bw day^-1^ E_2_ group were significantly enriched in 43 pathways including “drug metabolism-cytochrome P450”, “proteoglycans in cancer”, “estrogen signaling pathway”, and “endometrial cancer”. DEGs in the 3 mg kg^-1^ bw day^-1^ PhOP group were significantly enriched in “PPAR signaling pathway”, “fat digestion and absorption”, “cytokine-cytokine receptor interaction”, and “asthma”. DEGs in the 30 mg kg^-1^ bw day^-1^ PhOP group were significantly enriched in 41 pathways, most of which were consistent with those of the E_2_ group. DEGs in the 3 mg kg^-1^ bw day^-1^ PeOP group were significantly enriched in five pathways, including "type I diabetes mellitus" and "autoimmune thyroid disease". DEGs in the 30 mg kg^-1^ bw day^-1^ PeOP group were significantly enriched in 11 pathways, including "complement and coagulation cascades", "intestinal immune network for IgA production", and “PPAR signaling pathway”. Figure 5 illustrates the top pathways with the smallest *p*-values that the DEGs were significantly enriched in. Only 0.4 mg kg^-1^ bw day^-1^ E_2_- and 30 mg kg^-1^ bw day^-1^ PhOP-treated groups were enriched in “estrogen signaling pathway”.

**Figure 6:**
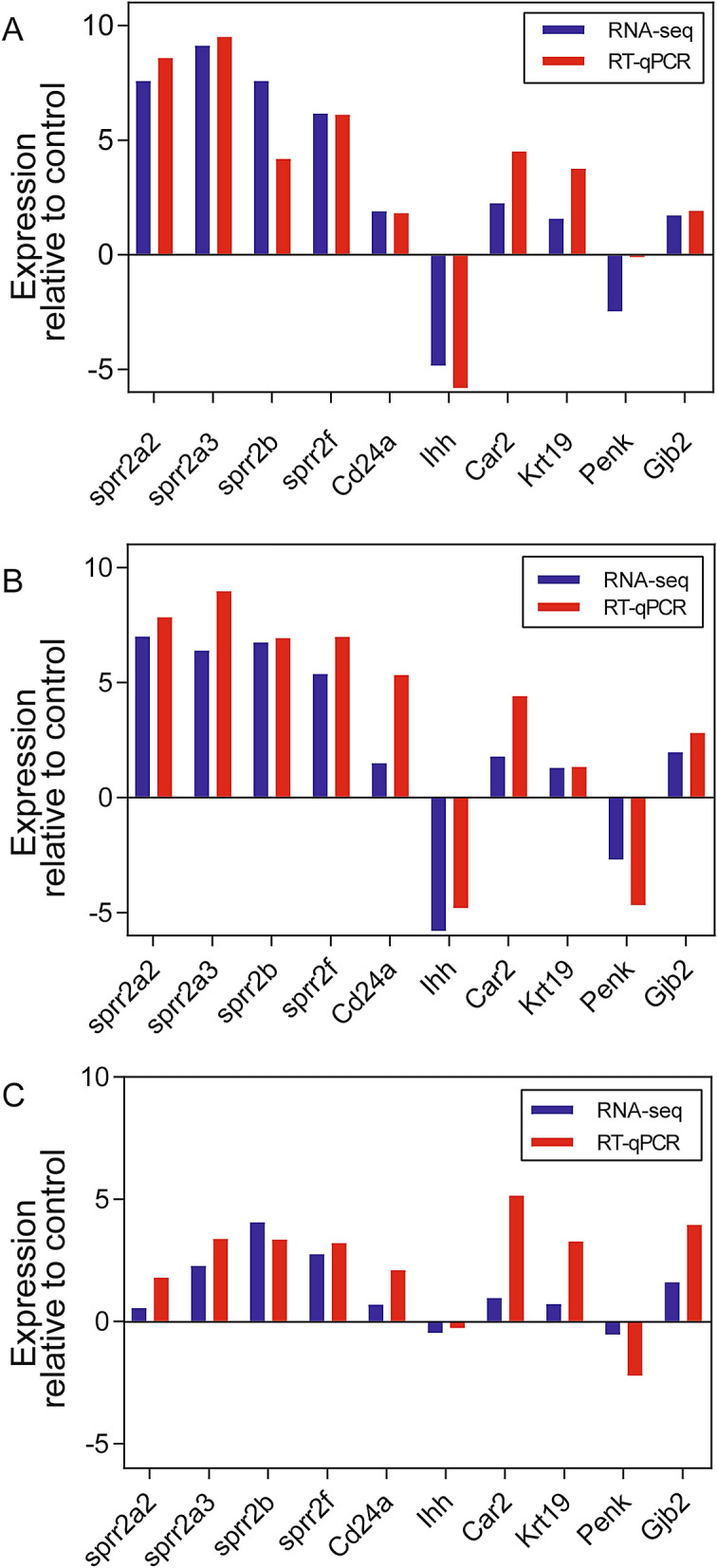
Validation of the expression of selected genes using real-time quantitative reverse transcriptase PCR (RT-qPCR). Gene symbols of the selected genes are positioned below the *x*-axis. RT-qPCR validation of the mice uteri treated with **(A)** 0.4 mg kg^-1^ bw day^-1^ 17*β*-estradiol (E_2_); **(B)** 30 mg kg^-1^ bw day^-1^ *p*-phenoxyphenol (PhOP); and **(C)** 30 mg kg^-1^ bw day^-1^ *p*-pentyloxyphenol (PeOP).

To validate the RNA-seq results, expressions of 10 estrogen-responsive genes were measured by RT-qPCR. The results demonstrated good consistency between the RNA-seq and RT-qPCR data (Fig. 6).

### PeOP and PhOP interactions with human ERα

**Figure 7:**
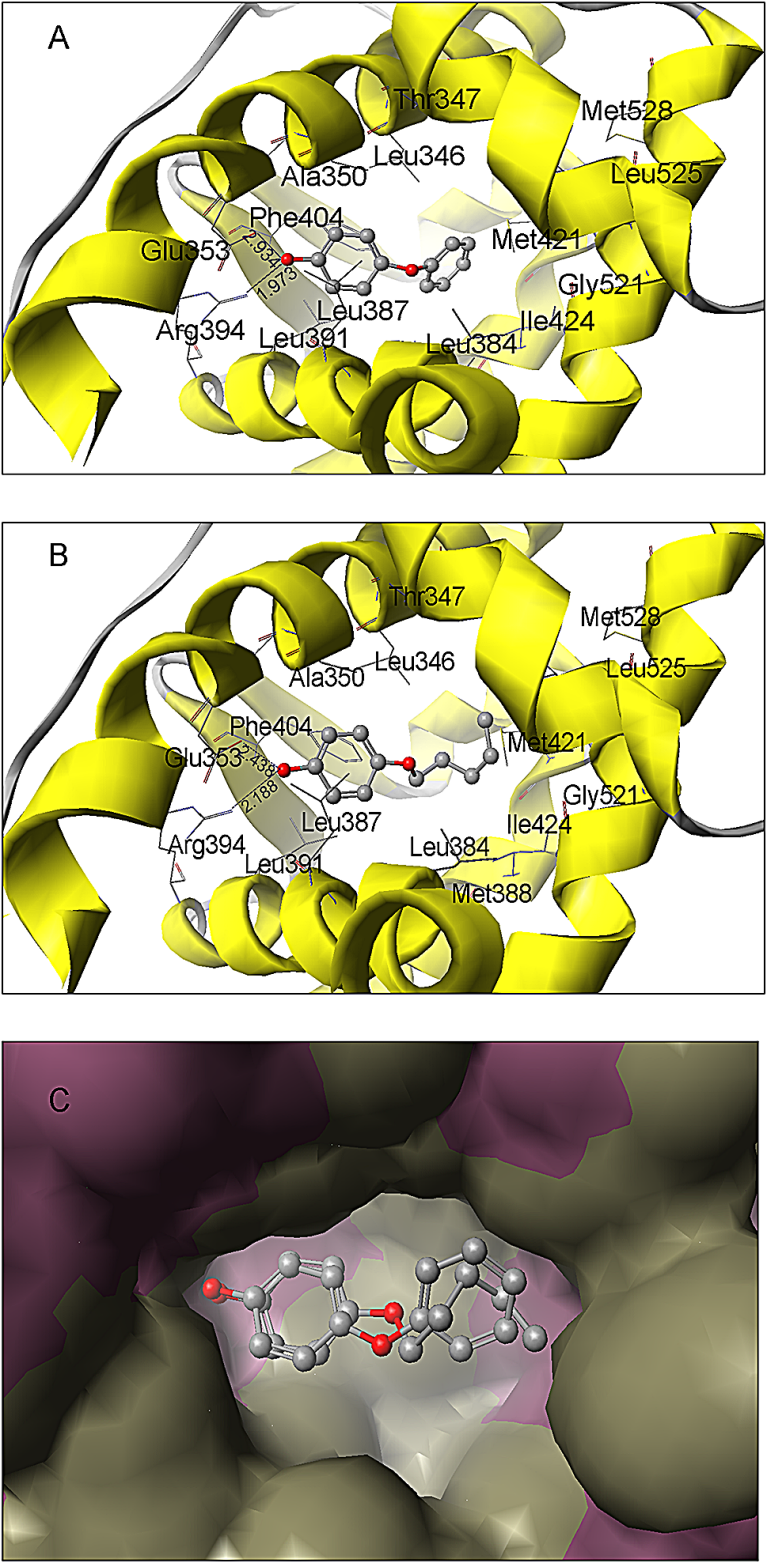
Molecular docking analysis of *p*-phenoxyphenol (PhOP) and *p*-pentyloxyphenol (PeOP) in the active site of hERα (PDB ID 1GWR). Docking results of **(A)** PhOP and **(B)** PeOP; **(C)** simulated binding position of PhOP and PeOP in the hERα active site. The dotted lines indicate the hydrogen bonds formed between the ligands and active site of hERα.

Molecular docking provides a clear understanding of the interactions between chemicals and hERα^43–45^. The predicted positions and binding poses of PhOP and PeOP in the active site of the hERαLBD template (PBD ID 1GER) are illustrated in Fig. 7. The PMF values of PhOP and PeOP were − 58.68 and − 52.67 kcal mol^-1^, respectively, indicating that both chemicals could fit well into hERα. The main interactions between the chemicals and hERα active sites were hydrogen bonds and hydrophobic interactions. The phenolic hydroxyl groups in PhOP and PeOP formed hydrogen bonds with the side chains of Glu353 and Arg349 in the active pocket, while their hydrophobic groups formed hydrophobic interactions with the hydrophobic amino acids (Leu346, Leu387, Leu391, Phe404, Met 421, Ile424, and Leu525) in the active pocket (Fig. 7A,B). Figure 7C depicts the postures of PhOP and PeOP positions in the hERα active pocket.

## Conclusions

In conclusion, PhOP presented marked estrogenic activity in vivo. It significantly increased the uterine weights of immature mice at doses of 30 and 300 mg kg^-1^ bw day^-1^ and induced the expression of the estrogen-response genes in mice uteri. In contrast, PeOP displayed a weak uterotrophic effect; however, it significantly affected the expression of the estrogen-response genes in mice uteri.

## Materials and methods

### Chemicals

PeOP (> 97%), PhOP (> 98%), and E_2_ (> 98%) were purchased from Aladdin Reagent Co., Ltd. (Shanghai, China).

### Immature mice uterotrophic assays

Female CD-1 mice aged 20 postnatal days (PND) were purchased from Beijing Vital River Laboratory Animal Technology Co., Ltd. (Beijing, China). All the animal studies were approved by the Institutional Animal Care and Use Committee of Peking University and carried out according to the Guidelines for Animal Experiments of the university, which were in line with the ethical guidelines for the use of experimental animals in China. After acclimatization, the mice were assigned to treatment and control groups using a completely randomized design (n = 8). All the animals were kept in identical environments with continuous food and water availability.

PhOP and PeOP were dissolved in peanut oil (vehicle) to prepare dosages of 3, 30, and 300 mg kg^-1^ bw day^-1^, respectively. Mice treated with peanut oil only were used as the peanut oil vehicle control, while those treated with 0.4 mg kg^-1^ bw day^-1^ E_2_^43,46^ were used as the positive control. The mice were treated for three days by oral gavage from PND 21. The mice in each cage were marked by shaving hairs on different parts of the body, and each group was treated by oral gavage in turn, where by in each turn only one mouse in each group was treated. Each group of mice was then weighed and sacrificed by cervical dislocation after 24 h from the last treatment. The uteri were isolated, weighed, and frozen in liquid nitrogen until use for next-generation transcriptome sequencing (RNA-seq). The relative uterine weight, represented as the uterine-to-final-body weight (bw) ratio, was determined to evaluate the uterotrophic activities of the chemicals. To reduce the risk of bias in the data collection, three researchers were responsible for different experimental steps: the first researcher was responsible for catching mice and recording data; the second was responsible for body weighing, killing by cervical dislocation, and dissecting the abdominal cavity; the third was responsible for cutting, blotting, and weighting of the uteri. The latter two researchers were blind to the group of the mice.

### Next-generation sequencing-based transcriptome analysis

The uteri of the immature CD-1 mice from the 0.4 mg kg^-1^ bw day^-1^ E_2_, 3 mg kg^-1^ bw day^-1^ PhOP, 30 mg kg^-1^ bw day^-1^ PhOP, 3 mg kg^-1^ bw day^-1^ PeOP, 30 mg kg^-1^ bw day^-1^ PeOP, and peanut oil vehicle control groups were selected for RNA-seq. The total RNA was extracted from the uteri of each group (n = 3) using TRIzol reagent (Invitrogen, Carlsbad, CA, USA) and the concentrations were measured using a NanoDrop 2000 spectrophotometer (Thermo Scientific, Waltham, MA, USA). RNA contamination and degradation were estimated by agarose gelation gel electrophoresis, the integrity of which was checked using an Agilent 2100 bioanalyzer (Agilent Technologies, Inc., Santa Clara, CA, USA). The Hiseq-PE150 sequencing platform was used to construct a raw data library with a data volume of 6G. The total RNA from each pool of samples (a mixture from three animals) was used for high-throughput sequencing according to the Illumina transcriptome sequencing method (Illumina, San Diego, CA, USA; Beijing Novogene Co., Ltd.).

RNA-seq analysis was performed by CLC Genomics Workbench 12 with reference gene model annotations (*Mus musculus* GRCm38. p6). The number of readings per kilobase length from a gene per million readings (RPKM) was as the relative transcript levels for comparison. The Baggerley test^47^ was used to statistically evaluate the RPKM value of each gene to determine differential gene expression between groups. Genes were considered as differentially expressed genes (DEGs) at *P* < 0.05 and an absolute value of fold changes (FC) > 2. The Database for Annotation, Visualization, and Integrated Discovery (DAVID; https://david.abcc.ncifcrf.gov/) was used for the Gene Ontology (GO) and Kyoto Encyclopedia of Genes and Genomes (KEGG) pathway analyses. R 3.6.1 was primarily used for data visualization. The final RNA-Seq data has been deposited in the NCBI GEO database (GSE151242).

### Real-time quantitative reverse transcriptase PCR (RT-qPCR)

RT-qPCR with SYBR green fluorescence detection was performed to verify the gene expression using an Agilent Mx3005P real-time PCR machine (Agilent Technologies). Ten estrogen responsive genes were selected for validation. The primers were designed using Primer Express Software v3.0 (Applied Biosystems, USA) and are listed in Table 1. Actin beta (*Actb*) was used as an endogenous control to normalize the data. The 2^−ΔΔCt^ method was used to determine the relative gene expression levels^48^.

**Table 1:** Primers used for real-time quantitative reverse transcriptase PCR (RT-qPCR).

Gene symbol	Forward primer (5′ → 3′)	Reward primer (5′ → 3′)
*Actb*	AGATGACCCAGATCATGTTTGAGA	CACAGCCTGGATGGCTACGT
*Sprr2b*	GTGTCCACCCAAGAATAAATGAG	AGGACAGGCGTTCAAAGGAG
*Sprr2a2*	GGTCACTGCTGTTTCATTTCCT	ATTAGACCATCACCAAAGGGG
*Sprr2a3*	TCTCCACCCTTCATCCTCCAT	GAGATCAGCCTGAGAGCAATGC
*Sprr2f*	ATGGGTCTTGTTCCATTGTTCA	AACAGTAACAACTACCCTGCTCAAG
*Car2*	TCTGCTCTGCCCCAATCAC	TCTGGTCCGTTGTGCTTGCT
*Cd24a*	CTGCTTCTGGCACTGCTCCTA	TTACCGGGAAACGGTGCAA
*Gjb2*	GACACAGTGCCAACCATCCA	ACCGTGAGCCAGATCTTTCCA
*Ihh*	GGCTTCGACTGGGTGTATTACG	CGGCCGAATGCTCAGACTT
*Krt19*	TGACTTCAGAACCAAGTTTGAGACA	GCGCAGGCCGTTGATGT
*Penk*	AGCCAGGACTGCGCTAAATG	GTGTGCACGCCAGGAAATTG

### Molecular docking

Scigress software (Ultra Version 3.4.0; Fujitsu Ltd., Tokyo, Japan) was used for in silico molecular docking analysis according to previously reported methods (Zhang et al. 2017). The three-dimensional (3D) protein structure of the hERα LBD in complex with TIF2 NRBox3 (PDB ID: 1GWR) was downloaded from the Protein Data Bank (PDB, https://www.rcsb.org/pdb). The 3D structure was reduced to a monomeric structure. Water and small molecules (except those that were considered important to the receptor pocket) were deleted and the refined protein structure was preserved. The molecular structures with PhOP and PeOP were then drawn, hydrogenated, and energy optimized by the PM3 mode of the Scigress-integrated procedures. The hERα active site was selected for molecular docking with PhOP and PeOP, whereby the ligand was set to be flexible and the active site was set to be rigid. The grid was evaluated using a 15 × 15 × 15 Å grid frame with the grid spacing 0.375 Å. The program was set to 60,000 generations with an initial population size of 50, accuracy of 5, a crossover rate of 0.8, and a mutation rate of 0.2. The potential of mean force (PMF), a knowledge-based approach that extracts pairwise atomic potentials from the structure information of the receptor-ligand complexes, was used to evaluate the binding potential of the chemicals with hERα^49^.

### Data analysis

The data were analyzed using the statistical program SPSS (v.18.0; Chicago, IL, USA) and presented as means and standard errors of mean (SEMs) unless otherwise indicated. The group differences were evaluated using one-way analysis of variance and Fisher's least significant difference tests, with *P* < 0.05 considered of statistical significance (Supplementary information).

## Supplementary information

Supplementary Information.
