# Supplementary material for: In vivo estrogenicity of p-phenoxyphenol and p-pentyloxyphenol
Source: Sci Rep. 2020 Oct 14;10:17305. doi: 10.1038/s41598-020-73271-1 (PMC7560878; doi:10.1038/s41598-020-73271-1)
Supplement: Supplementary file 1 — Supplementary Information. [file 41598_2020_73271_MOESM1_ESM.docx]

*In vivo* estrogenicity of *p*-phenoxyphenol and *p*-pentyloxyphenol

Yue Wang, Han Xiao, Lei Yang, Xiaojing Jia, Xuan Guo, Zhaobin Zhang*

* Corresponding author

Tel.: +86 10 62755168; Fax: +86 10 62755168

E-mail: zhangzb@pku.edu.cn

College of Urban and Environmental Sciences, MOE Laboratory for Earth Surface Processes, Peking University, Beijing 100871, China.

Supplementary table

Table S1. Total DEGs in the 17*β*-estradiol (E_2_)-, PhOP-, and PeOP-treated groups

| Gene Symbol |
| --- |
| *0610040J01Rik*, *1500015A07Rik*, *1500015O10Rik*, *1600014C10Rik*, *1600023N17Rik*, *2210407C18Rik*, *2410006H16Rik*, *2700081O15Rik*, *2810459M11Rik*, *4833423E24Rik*, *6030408B16Rik*, *6330403K07Rik*, *9530014B07Rik*, *A2m*, *A330049N07Rik*, *A4galt*, *Aard*, *Abcb1a*, *Abcc9*, *Acaa2*, *Acod1*, *Acot1*, *Acsf2*, *Acss1*, *Adamts7*, *Add2*, *Adgra2*, *Adra2c*, *Afap1l1*, *Agr2*, *Ahcy*, *Ahcyl2*, *Ahsg*, *AI506816*, *AI661453*, *Ajap1*, *Akr1c13*, *Alb*, *Alcam*, *Aldh1a3*, *Aldh7a1*, *Aldoc*, *Alox15*, *Alpl*, *Ambp*, *Angptl2*, *Angptl7*, *Ano1*, *Anxa8*, *Aoc1*, *Aox3*, *Ap1s3*, *Apcdd1*, *Apoa1*, *Apoa2*, *Apobec2*, *Apoc3*, *Apol7a*, *Aqp1*, *Aqp5*, *Arg1*, *Arhgef17*, *Arl4c*, *Arnt2*, *Atp10b*, *Atp1a2*, *Atp2a3*, *Atp2b2*, *AU021092*, *Avil*, *Axin2*, *Axl*, *Barx2*, *BC023719*, *BC048679*, *Bcat1*, *Bcl2l15*, *Bex1*, *Bhlhe40*, *Bicc1*, *Bmp4*, *Bmp7*, *Bnipl*, *Bpifb5*, *C130074G19Rik*, *C1qc*, *C1qtnf12*, *C1qtnf4*, *C1ra*, *C1rb*, *C1s1*, *C1s2*, *C2cd4a*, *C3*, *Cacng7*, *Cadm4*, *Calb1*, *Calcoco1*, *Cap2*, *Capn5*, *Car2*, *Car9*, *Cavin2*, *Cblc*, *Cbr2*, *Cbs*, *Ccl11*, *Ccl28*, *Ccl8*, *Ccnd2*, *Cd248*, *Cd24a*, *Cd302*, *Cd74*, *Cd93*, *Cdh16*, *Cdh5*, *Cdkn1c*, *Ceacam1*, *Ceacam2*, *Cebpa*, *Cfb*, *Cfi*, *Cfp*, *Cftr*, *Chil1*, *Chil4*, *Chst8*, *Cited4*, *Ckmt1*, *Clca1*, *Cldn10*, *Cldn2*, *Cldn5*, *Cldn8*, *Clec3b*, *Clip3*, *Cndp2*, *Coch*, *Col18a1*, *Col23a1*, *Col6a4*, *Colec12*, *Cox7b*, *Cpe*, *Cped1*, *Cpxm2*, *Crabp1*, *Crabp2*, *Crtac1*, *Ctse*, *Ctsf*, *Cwc22*, *Cxcl12*, *Cxcl14*, *Cxcl15*, *Cxcl17*, *Cyb5b*, *Cyb5r3*, *Cygb*, *Cyp21a1*, *Cyp27a1*, *Cys1*, *D630039A03Rik*, *Dact1*, *Dact2*, *Dbp*, *Ddr2*, *Dhcr24*, *Dhrs9*, *Dkk2*, *Dkk3*, *Dlx5*, *Dlx6*, *Dlx6os1_1*, *Dmbt1*, *Dsp*, *Dtd1*, *Dync2li1*, *Ebf4*, *Edn3*, *Efemp1*, *Efnb3*, *Efs*, *Egfl6*, *Egflam*, *Ehf*, *Eid1*, *Elf3*, *Emb*, *Enpp2*, *Epcam*, *Ephx1*, *Epsti1*, *Esam*, *Etnppl*, *Etv5*, *Evpl*, *Exosc6*, *F2r*, *Fabp4*, *Fam117a*, *Fam181b*, *Fam184b*, *Fam20c*, *Fam213a*, *Fam3b*, *Fam84a*, *Fasn*, *Fcgbp*, *Fer1l4*, *Fmo1*, *Fmo2*, *Fndc1*, *Fndc5*, *Fos*, *Foxl2*, *Fst*, *Fuom*, *Fut4-ps1*, *Fyb2*, *Fyn*, *Fzd10*, *Fzd2*, *Gabrp*, *Galm*, *Galnt12*, *Galnt15*, *Galnt3*, *Gap43*, *Gc*, *Gdf10*, *Gdf15*, *Gdf7*, *Gdpd3*, *Gfra2*, *Ghrh*, *Gimap4*, *Gjb2*, *Gli1*, *Glis2*, *Glul*, *Gm10039*, *Gm10238*, *Gm10320*, *Gm10499*, *Gm11127*, *Gm12473*, *Gm13443*, *Gm14005*, *Gm15772*, *Gm1821*, *Gm21451*, *Gm21596*, *Gm266*, *Gm26880*, *Gm26892*, *Gm28309*, *Gm28592*, *Gm3336*, *Gm34030*, *Gm42031*, *Gm43305*, *Gm4366*, *Gm45397*, *Gm46223*, *Gm47136*, *Gm4737*, *Gm47754*, *Gm48898*, *Gm49425*, *Gm49450*, *Gm5294*, *Gm5514*, *Gm5611*, *Gm6563*, *Gm7879*, *Gm8730*, *Gm8909*, *Gm9008*, *Gm9118*, *Gm9770*, *Gmds*, *Gpc1*, *Gpc3*, *Gpd1*, *Gprc5b*, *Gpx2*, *Grb7*, *Greb1*, *Grem2*, *Grik5*, *Grp*, *Gsta4*, *Gstm1*, *Gstm2*, *Gstm7*, *Gstt1*, *Guca2b*, *Gulo*, *Gzma*, *Gzmb*, *Gzmc*, *Gzmd*, *Gzme*, *Gzmf*, *Gzmg*, *H1fx*, *H2-Aa*, *H2-Ab1*, *H2afy2*, *H2-D1*, *H2-Ea-ps*, *H2-Eb1*, *H2-K2*, *H2-T10*, *H2-T23*, *H2-T-ps*, *Hand2*, *Hdac11*, *Hdc*, *Hgfac*, *Hic1*, *Hlf*, *Hmga1b*, *Hmgcs2*, *Hopx*, *Hoxa10*, *Hoxa11*, *Hoxa11os*, *Hoxd10*, *Hoxd11*, *Hoxd9*, *Htra3*, *Id1*, *Id2*, *Id3*, *Id4*, *Ifit1bl1*, *Ifit3b*, *Ifitm1*, *Ifne*, *Igfbp3*, *Igfbp4*, *Igfbp5*, *Igha*, *Ighv1-37*, *Ighv1-53*, *Ighv1-69*, *Ighv1-72*, *Ighv3-6*, *Ighv5-6*, *Ighv9-1*, *Ighv9-4*, *Igkc*, *Igkv10-96*, *Igkv12-89*, *Igkv15-103*, *Igkv17-127*, *Igkv1-88*, *Igkv3-7*, *Igkv4-72*, *Igkv6-20*, *Igkv8-28*, *Igkv9-124*, *Iglc1*, *Iglv1*, *Ihh*, *Il17d*, *Il17rb*, *Il1f6*, *Il20rb*, *Il2rb*, *Inhbb*, *Inmt*, *Irs1*, *Islr*, *Itgam*, *Itm2a*, *Jag2*, *Jam2*, *Jchain*, *Kank4*, *Kap*, *Kcnj8*, *Kcnn4*, *Kctd14*, *Khk*, *Klf15*, *Klf2*, *Klf4*, *Klf9*, *Klk1*, *Klk11*, *Klk1b21*, *Kpna2*, *Krt14*, *Krt15*, *Krt19*, *Krt20*, *Krt23*, *Krt5*, *Krt7*, *Krt8*, *Krt83*, *Krt84*, *Krt87*, *Lad1*, *Lamb3*, *Lamc3*, *Lcn2*, *Ldhb*, *Ldlr*, *Lepr*, *Lgals3*, *Lhfp*, *Lipm*, *Lrig1*, *Lrp2*, *Lrrc17*, *Lrrc26*, *Lsp1*, *Ltbp4*, *Ltf*, *Ly6c1*, *Ly6d*, *Lypd2*, *Lyz2*, *Lzts3*, *Mal2*, *Malat1*, *Mall*, *Mamdc2*, *Maoa*, *Maob*, *Mapk13*, *Mapk8ip1*, *Mapkapk3*, *Matn2*, *Mcub*, *Mdk*, *Mest*, *Mfap4*, *Mgat3*, *Mgll*, *Mill2*, *Misp*, *Mkrn2os*, *Mllt6*, *Mlph*, *Mme*, *Mmp2*, *Mmp7*, *Mmrn2*, *Mogat1*, *Morn4*, *Moxd1*, *Mpzl2*, *Msx1*, *Msx2*, *Mt2*, *Mturn*, *Muc1*, *Muc20*, *Muc4*, *Mup3*, *Myb*, *Mybpc1*, *Myc*, *Mycn*, *Myh14*, *Nab2*, *Napsa*, *Nbl1*, *Ndp*, *Nectin4*, *Nfe2l3*, *Nfic*, *Ngfr*, *Nid1*, *Nid2*, *Nipsnap1*, *Nkd2*, *Nkg7*, *Nop53*, *Noxa1*, *Npl*, *Nr1d1*, *Nr1d2*, *Nrep*, *Nrgn*, *Nrtn*, *Nsg2*, *Nt5e*, *Ntn4*, *Ntn5*, *Ntrk2*, *Oaf*, *Oit1*, *Olfml2a*, *Omp*, *Osr2*, *Ovgp1*, *Pabpc1l2b-ps*, *Padi1*, *Padi2*, *Padi4*, *Palm*, *Pamr1*, *Pcolce2*, *Pcx*, *Pdcd4*, *Pdgfra*, *Pdgfrb*, *Pdk2*, *Pdpn*, *Penk*, *Per1*, *Per2*, *Per3*, *Perp*, *Phgdh*, *Phldb1*, *Pi16*, *Pianp*, *Pigr*, *Pim3*, *Pkdcc*, *Pla2g10*, *Pla2g2e*, *Pla2g5*, *Pla2g7*, *Plat*, *Plekha6*, *Plekhs1*, *Plet1*, *Pltp*, *Porcn*, *Ppp1r1b*, *Ppp2r2b*, *Ppp2r2c*, *Prap1*, *Prg4*, *Procr*, *Prodh*, *Pros1*, *Prss22*, *Prss32*, *Psca*, *Ptch1*, *Ptgs2*, *Pth1r*, *Ptk7*, *Ptpn5*, *Ptpru*, *Rab11fip4*, *Rab15*, *Rab25*, *Rab32*, *Radil*, *Ramp3*, *Rasd2*, *Rasip1*, *Rassf10*, *Rbbp9*, *Rbm24*, *Reck*, *Rem1*, *Resf1*, *Retnla*, *Rev3l*, *Rftn1*, *Rgl3*, *Rgs2*, *Rhob*, *Rilpl1*, *Rn7s1*, *Rnaset2b*, *Rnf186*, *Rnf208*, *Rp9*, *Rpl30*, *Rps2*, *Rps24-ps3*, *Rps2-ps13*, *Rps3a2*, *Rpsa-ps10*, *Rpsa-ps9*, *Rsph3a*, *Rspo1*, *Rspo3*, *Rtl8c*, *S100a8*, *S100a9*, *S1pr3*, *Sbspon*, *Scara3*, *Scara5*, *Scarb1*, *Scd1*, *Scin*, *Scube1*, *Scx*, *Sdc2*, *Sdc4*, *Sdcbp2*, *Selenop*, *Sema5a*, *Serpina3k*, *Serpinb11*, *Serpine2*, *Sfn*, *Sfrp2*, *Sftpd*, *Shisa2*, *Siglec15*, *Slc14a1*, *Slc16a14*, *Slc16a2*, *Slc22a3*, *Slc23a4*, *Slc25a48*, *Slc26a4*, *Slc27a3*, *Slc28a3*, *Slc29a1*, *Slc2a3*, *Slc31a2*, *Slc34a2*, *Slc37a1*, *Slc40a1*, *Slc44a4*, *Slc5a1*, *Slc5a11*, *Slc5a9*, *Slc6a2*, *Slc6a6*, *Smarca2*, *Smoc2*, *Smpd3*, *Smpdl3a*, *Snai1*, *Sned1*, *Sorbs3*, *Sord*, *Sox17*, *Sp5*, *Sparcl1*, *Spink1*, *Spink12*, *Spock2*, *Spp1*, *Sprr1a*, *Sprr2a2*, *Sprr2a3*, *Sprr2b*, *Sprr2d*, *Sprr2f*, *Sprr2g*, *Spry1*, *Srgn*, *St14*, *Stc2*, *Steap1*, *Steap3*, *Steap4*, *Stra6*, *Sulf1*, *Sult1d1*, *Sun2*, *Syn2*, *Syt8*, *Tacstd2*, *Tbc1d16*, *Tbx2*, *Tbx3*, *Tcf21*, *Tcf7*, *Tef*, *Tenm3*, *Tent5b*, *Tfap4*, *Tfcp2l1*, *Tfrc*, *Tgfb3*, *Tgfbi*, *Thbd*, *Thrsp*, *Thy1*, *Tie1*, *Timp3*, *Tjp3*, *Tmem132c*, *Tmem30b*, *Tmem45b*, *Tmem54*, *Tmprss11d*, *Tmprss11g*, *Tnfrsf19*, *Tnfsf12*, *Tnnt1*, *Tns2*, *Tnxb*, *Tpm3-rs7*, *Tppp3*, *Tpsb2*, *Trf*, *Trpv6*, *Tsc22d3*, *Tshz1*, *Tspan1*, *Tspan4*, *Tspan8*, *Tst*, *Ttc9*, *Twist2*, *Txnip*, *Tyrobp*, *Ugt1a1*, *Ugt1a7c*, *Unc5cl*, *Uox*, *Upk1a*, *Upk3b*, *Upk3bl*, *Urah*, *Usp2*, *Vegfa*, *Vim*, *Vit*, *Vwa2*, *Wee1*, *Wfdc1*, *Wfdc15b*, *Wfdc2*, *Wfikkn2*, *Wif1*, *Wnt11*, *Wnt16*, *Wnt6*, *Wnt7a*, *Wnt7b*, *Xcl1*, *Xist*, *Ypel3*, *Zbtb20*, *Zbtb4*, *Zfas1*, *Zfyve21* |
